# Supplementary material for: Addressing a Rule of Thumb: Modeling the Effects of Meteorological Conditions on Prescription of Antimicrobials in Aquaculture
Source: Microbiol Spectr. 2022 Sep 19;10(5):e01752-22. doi: 10.1128/spectrum.01752-22 (PMC9603815; doi:10.1128/spectrum.01752-22)
Supplement: Supplemental file 1 — Fig. S1 to S4; Table S1. Download spectrum.01752-22-s0001.pdf, PDF file, 0.2 MB [file spectrum.01752-22-s0001.pdf]

Supplementary material

Supplementary figures 1-4

Supplementary table 1

A separate file containing all statistical analyses is available upon request

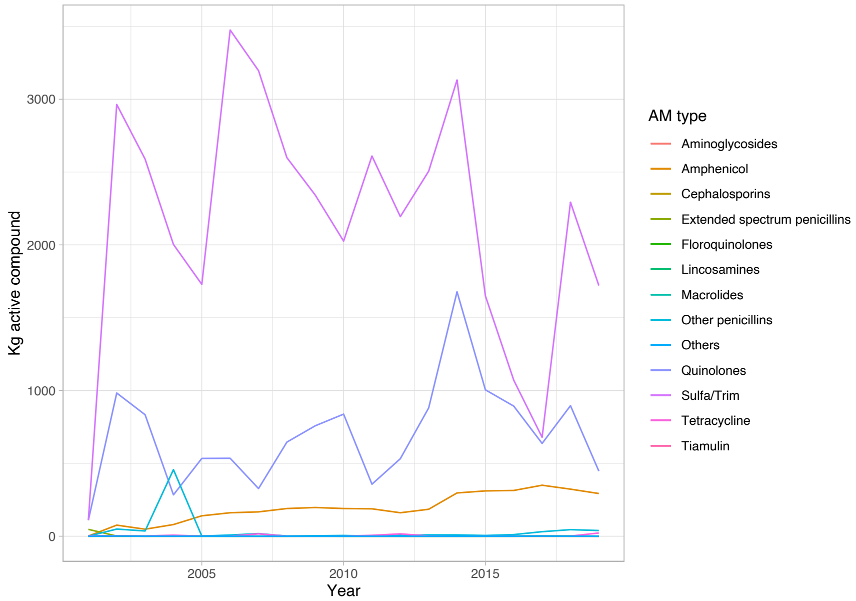

Supplementary figure 1: Total antimicrobial use in Danish aquaculture by type.

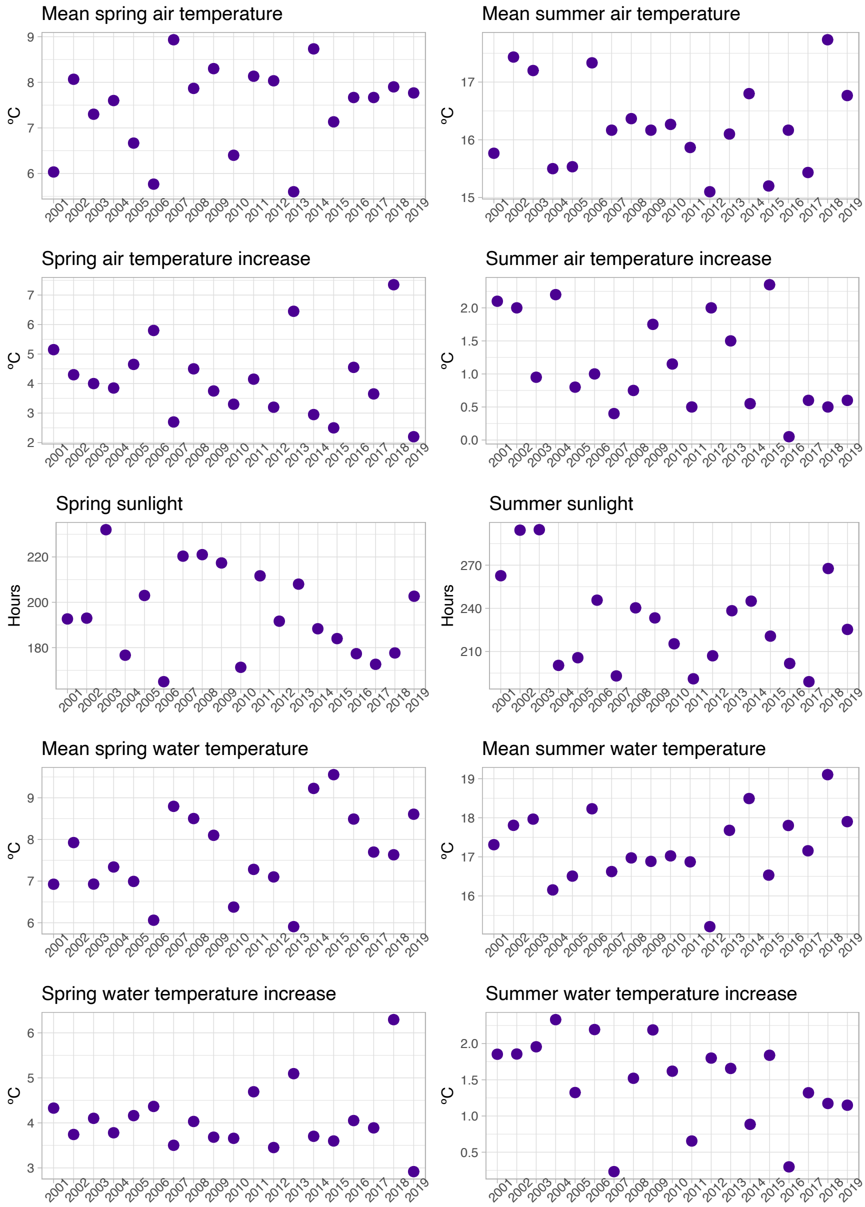

Supplementary figure 2: Meteorological variables included in the present study. See materials and methods section for data sources and processing. The data plotted here are also shown in tabular form in supplementary table 1. . It should be noted, that for May-August 2002 and for March-August 2003, the available data for recorded hours of sunlight are specific to the Copenhagen, Denmark area.

Commented [AMB1]: Det er en relevant oplysning, men måske var det bedre at levere den som en note til en figur eller lign.? I betragtning af hvor lille en effekt det må forventes at bidrage med, synes jeg et de to linjer hér måske er lidt malplacerede.

Commented [KRV2R1]: Jeg kan godt se hvad du mener. I så fald vil jeg gerne flytte den til figurteksten til DMI-data, samt til tabellen over samme.

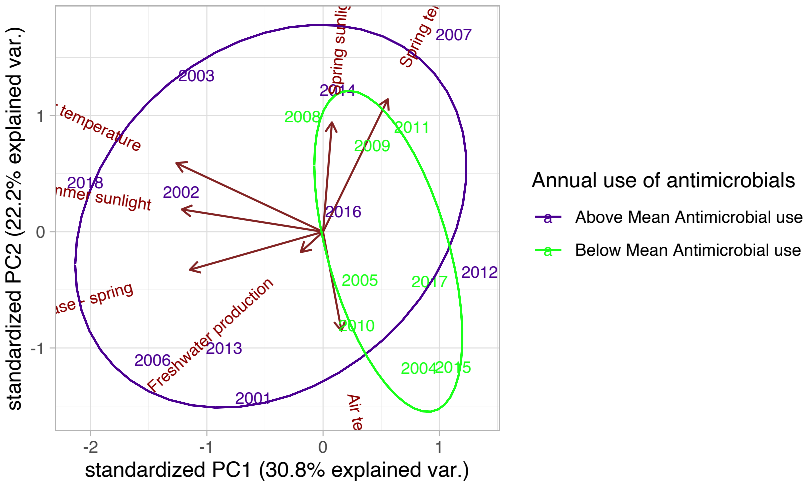

Supplementary figure 3: Principal component analysis plot of land-based production model parameters. Originating from an arbitrary center, arrows indicate the loading of each model parameter relative to the variation along PC1 and PC2. Data for each year is indicated within the plot and color coded, with purple and black years having higher and lower than mean annual AM use, respectively.

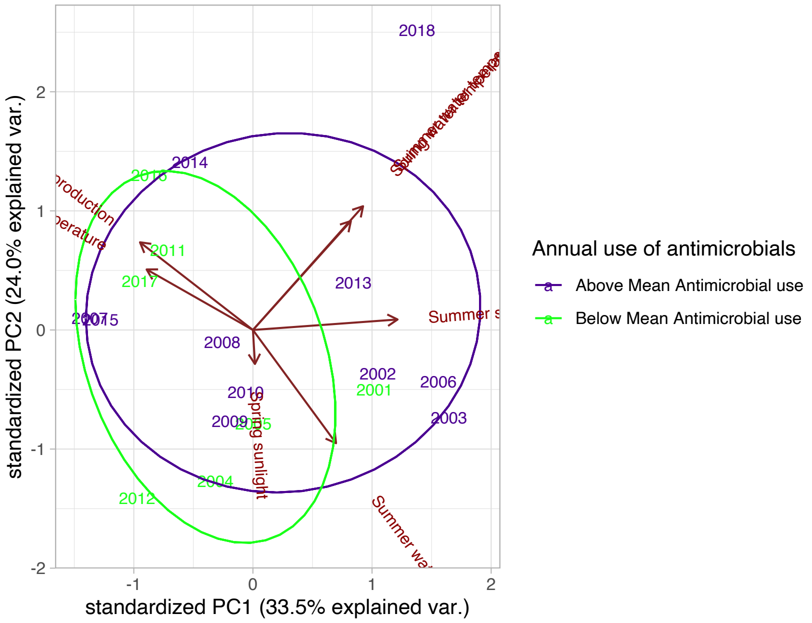

Supplementary figure 4: Principal component analysis plot of Marine production model parameters. Originating from an arbitrary center, arrows indicate the loading of each model parameter relative to the variation along PC1 and PC2. Data for each year is indicated within the plot and color coded, with purple and black years having higher and lower than mean annual AM use, respectively.

Year	Spring air temperature (°C)	Spring air temperature increase °C)	Summer air temperature (°C)	Summer air temperature increase (°C)	Spring sunlight (h)	Summer sunlight (h)	Mean spring water temperature (°C)	Mean summer water temperature (°C)	Spring water temperature increase (°C)	Summer water temperature increase (°C)
2001	6.0	5.2	15.8	2.1	192.7	262.7	6.92	17.31	4.33	1.85
2002	8.1	4.4	17.4	2.0	193.0	294.3	7.92	17.81	3.74	1.86
2003	7.3	4.0	17.2	1.0	232.0	294.7	6.93	17.97	4.10	1.96
2004	7.6	3.9	15.5	2.2	176.7	200.3	7.34	16.15	3.78	2.33
2005	6.7	4.7	15.5	0.8	203.0	205.7	6.99	16.51	4.16	1.32
2006	5.8	5.8	17.3	1.0	165.0	245.7	6.06	18.23	4.37	2.19
2007	8.9	2.7	16.2	0.4	220.3	193.0	8.79	16.62	3.50	0.23
2008	7.9	4.5	16.4	0.8	221.0	240.3	8.50	16.97	4.03	1.52
2009	8.3	3.9	16.2	1.8	217.3	233.3	8.10	16.88	3.68	2.19
2010	6.4	3.3	16.3	1.2	171.3	215.3	6.38	17.02	3.66	1.62
2011	8.1	4.4	15.9	0.5	211.7	191.0	7.28	16.87	4.69	0.65
2012	8.0	3.5	15.1	2.0	191.7	207.0	7.10	15.21	3.45	1.80
2013	5.6	6.5	16.1	1.5	208.0	238.3	5.91	17.68	5.09	1.66
2014	8.7	3.0	16.8	0.6	188.3	245.0	9.22	18.49	3.70	0.88
2015	7.1	2.5	15.2	2.4	184.0	220.7	9.56	16.53	3.60	1.84
2016	7.7	4.7	16.2	0.1	177.3	201.7	8.49	17.80	4.05	0.30
2017	7.7	3.8	15.4	0.6	172.7	189.0	7.70	17.16	3.89	1.32
2018	7.9	7.4	17.7	0.5	177.7	267.7	7.63	19.10	6.29	1.17
2019	7.8	2.2	16.8	0.6	202.7	225.3	8.61	17.90	2.92	1.15

Supplementary table 1: Summary of the meteorological data foundation. It should be noted, that for May-August 2002 and for March-August 2003, the available data for recorded hours of sunlight are specific to the Copenhagen, Denmark area.
